# Process evaluation of a behaviour change approach to improving clinical practice for detecting hereditary cancer

**DOI:** 10.1186/s12913-019-3985-5

**Published:** 2019-03-20

**Authors:** Janet C. Long, Teresa Winata, Deborah Debono, Kim-Chi Phan-Thien, Christine Zhu, Natalie Taylor

**Affiliations:** 1 Centre for Healthcare Resilience and Implementation Science, Australian Institute of Health Innovation, Faculty of Medicine and Health Sciences, Macquarie University, Sydney, NSW 2109 Australia; 2 Centre for Health Services Management, Faculty of Health, University of Technology, Sydney, NSW 2007 Australia; 3 St George and Sutherland Clinical School, University of New South Wales, Kensington, Sydney, NSW 2052 Australia; 4 Cancer Research Division, Cancer Council NSW, 153 Dowling St, Woolloomooloo, NSW 2011 Australia; 5 Faculty of Health Sciences, University of Sydney, Camperdown, Sydney, NSW 2006 Australia

**Keywords:** Theoretical domains framework implementation, Implementation, Hereditary cancer, Theory, Process evaluation

## Abstract

**Background:**

This retrospective process evaluation reports on the application of a 1-year implementation program to increase identification and management of patients at high risk of a hereditary cancer syndrome. The project used the *Theoretical Domains Framework Implementation* (TDFI) approach, a promising implementation methodology, used successfully in the United Kingdom to address patient safety issues. This Australian project run at two large public hospitals aimed to increase referrals of patients flagged as being at risk of Lynch syndrome on the basis of a screening test to genetic services. At the end of the project, the pathologists’ processes had changed, but the referral rate remained inconsistent and low.

**Methods:**

Semi-structured interviews explored participants’ perceptions of the TDFI approach and Health services researchers wrote structured reflections. Interview transcripts and reflections were coded initially against implementation outcomes for the various TDFI approach activities: acceptability, appropriateness, feasibility, value for time cost, and adoption. On a second pass, themes were coded around challenges to the approach.

**Results:**

Interviews were held with nine key project participants including pathologists, oncologists, surgeons, genetic counsellors and an administrative officer. Two health services researchers wrote structured reflections. The first of two major themes was ‘Theory-related challenges’, with subthemes of accessibility of theory underpinning the TDFI, commitment to that theory-based approach, and the problem of complexity. The second theme was ‘Practical challenges’ with subthemes of stakeholder management, navigating the system, and perceptions of the problem.

Health services researchers reflected on the benefits of bridging professional divides and facilitating collective learning and problem solving, but noted frustrations around clinicians’ time constraints that led to sparse interactions with the team, and lack of authority to effect change themselves.

**Conclusions:**

Mixed success of adoption as an outcome was attributed to the complexity and highly nuanced nature of the setting. This made identifying the target behaviour, a key step in the TDFI approach, challenging. Introduced changes in the screening process led to new, unexpected issues yet to be addressed. Strategies to address challenges are presented, including using an internal facilitator with a focus on applying a theory-based implementation approach.

**Electronic supplementary material:**

The online version of this article (10.1186/s12913-019-3985-5) contains supplementary material, which is available to authorized users.

## Background

Translating new technologies, novel diagnostic and screening procedures, and enhanced clinical understandings into routine clinical practice is a challenging endeavour [1–3]. Many different frameworks and models for achieving translation and implementation of research are used [4]. Process evaluations assess participants’ experience of using different approaches, evaluate the acceptability, feasibility, and influence of the context on implementation success [5]. Process evaluations complement and provide insight into results produced by primary outcome data, and inform plans for future projects [6].

We used the *Theoretical Domains Framework Implementation* (TDFI) [2] approach at two large Australian hospitals to increase identification and management of patients at high risk of a hereditary cancer condition, Lynch syndrome (LS) [7]. People with LS have a substantially higher than usual risk of bowel, endometrial and other cancers for themselves and their relatives [8]. If diagnosed early, carriers of LS can undergo increased surveillance to enable early detection, optimise treatment and decrease mortality for themselves and their relatives [9, 10]. Definitive diagnosis and appropriate risk management requires referral to a genetics service for a formal assessment but referral rates are known to be suboptimal [10], with an Australian study finding that less than half of CRC patients with high risk of LS were referred for genetic testing [11]. Although the incidence of LS is low, it is typical of a large number of hereditary conditions that genomic testing is increasingly able to identify. As genomic sequencing becomes more widely available, it is essential that a robust process be developed for multidisciplinary teams to identify and then manage patients and their families who are affected by these types of conditions.

The TDFI approach [1, 2] is grounded in behaviour change theory and is directed at changing an individual’s behaviour. As such it is useful for implementation problems where the choices or actions of individual health professionals are seen as the issue. The framework includes a consideration of physical and social contextual factors on the behaviour under scrutiny and may use system level changes to act as triggers or enablers of the desired new behaviour. It has been successfully used in a number of patient safety initiatives in the United Kingdom (UK) where safe practice was not consistently being enacted [1–3]. While the TDF itself [12] is used primarily to analyse barriers to individual’s behaviour change, the TDFI approach operationalises the TDF for application in clinical settings by providing an implementation framework that incorporates known drivers from implementation science [13] to prepare for change, analyse barriers, develop interventions and put them into practice. In the UK, the National Health Service has funded the Improvement Academy [14] to teach health care professionals how to use the TDFI. The project described here was the first to use the TDFI approach in an Australian context, and the first project to use it for a quality of care issue.

At the end of our project using the TDFI approach, we could demonstrate a number of beneficial practice changes in the process of referral, but we were unable to demonstrate a consistent change in the primary outcome: the referral rate of patients at high risk into genetic services. The aims of this paper are to conduct a process evaluation to: a) report on participants’ experience of using the TDFI approach, conducted at a time when this final outcome was not known, b) to reflect on the application of the approach in this context and reasons why it did not result in change in the primary outcome, and c) present proposed refinements to the TDFI approach to improve its application in future implementation research.

### The project

**Fig. 1:**
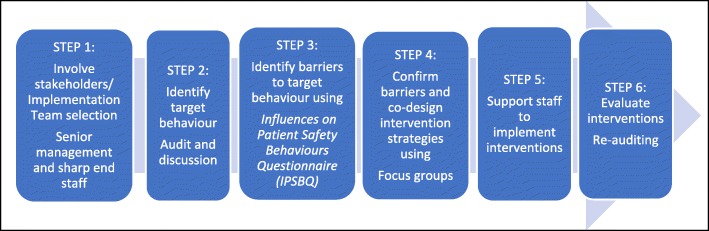
The six steps of the *Theoretical Domains Framework Implementation* approach

The *Achieving Behaviour Change for Identification and Management of Lynch Syndrome Project* was undertaken at two large Australian hospitals where, annually, over 200 patients have surgery to remove colorectal cancer tumours. Screening these tumours for LS resulted in around 20 high risk cases per year. At the time of the project, both the performance of the screening test and the response to high risk results were clearly acknowledged as best practice but were not mandated or articulated as hospital policy; that is, actions towards identification and management of at risk patients were at the discretion of individual health professionals. As such, the TDFI was seen to be well suited as a theoretical framework that targeted behaviour change in individuals. The project ran from April 2015 to June 2016 [15, 16]. The six steps of the TDFI process are shown in Fig. 1.

Activities for the project were the same for each hospital. Health services researchers worked with a multidisciplinary implementation team at each site (*n* = 8, 11 respectively) to map the process of referral, based on the patient journey, and a 12-month retrospective audit at each hospital (May 2014–April 2015) found referral rates were low (22% pooled results) [16]. Thirty-seven health professionals involved in the management of patients with colorectal cancer (CRC) completed the *Influences on Patient Safety Behaviours Questionnaire* to identify key barriers to referral according to the TDF domains [3]. Nineteen key healthcare professionals took part in TDF guided focus groups and interviews to discuss these results and, together with information from team meetings, process mapping, and audit, identified issues to be addressed. A mix of theoretically and intuitively derived interventions were developed and then implemented, led by members across the multidisciplinary team. Interventions included clarification of pathology report wording (led by pathologists), inclusion of a ‘genetic status’ field as a prompt on consultation summaries and GP letters (within individual medical/surgical treating teams), and training on hereditary cancers for rotating staff (led by clinical geneticists and counsellors). Towards the end of the project, initiated by a separate project, pathologists implemented a supplementary screening test to increase accuracy. This changed the process of referral considerably and streamlined the pathway. Referral rates of patients were monitored by continuing audits over the next year (until December 2016). Final referral rate for the year was 32% (7/22 patients) varying quarter to quarter from 17% (1/6) to 40% (2/5).

### Current work

This process evaluation assessed participants’ and researchers’ experience and perceptions of using the TDFI approach in this context. The evaluation sought to assess its success here and to inform future projects using the TDFI approach.

For the main project, referral data was the primary outcome of implementation success. Here we focussed on other related factors: acceptability, appropriateness, feasibility, adoption, and cost [17, 18]. Sustainability is another important measure of implementation success and will be monitored through ongoing audit. We also reflected on the influence of the context and reasons the main outcome indicator (referral rate) did not change consistently while the process of screening undoubtedly did.

## Methods

### Participants

We invited people who were involved in the project at one of the two hospitals to take part in a semi-structured interview to gather perceptions of the TDFI methodology, effectiveness of project activities, the experience of working with university-based health service researchers, contextual issues, and impacts and outcomes of the project. At this time, the final audit results (primary outcome data on referrals) were still being collected, so there was not yet any concrete evidence for success of the project.

We aimed to recruit up to 10 participants from different disciplines in order to get a range of perspectives. The identified people were members of the Project Team, or Implementation teams from Hospital A (*n* = 5) and Hospital B (*n* = 7) medical and radiology oncologists, surgeons, pathologists, genetic counsellors; consumer partners (*n* = 2) and administrative/liaison staff (*n* = 3). We invited potential interviewees by phone or email and provided a Participant Information Sheet. No inducements were offered.

### Interviews

Interview participants were asked open-ended questions designed to elicit comments on implementation outcomes: acceptability of the approach, its appropriateness in the oncology setting for addressing missed referral, its feasibility, cost, and extent of adoption. These outcomes have some overlapping features but all have utility in unpicking drivers and resistance to implementation projects [19]. Table 1 defines these implementation outcomes and summarises the interview questions asked to elicit information on each. The full interview schedule is provided in Additional file 1.

**Table 1:** Summary of implementation outcome measures, definitions, matched interview questions used to collect data

Measure	Definition	Interview questions explored:
Acceptability	Cognitive and emotional responses to the project [50].	• General perceptions of the project and whether their expectations of the project had been met.
Appropriateness	Compatibility with the individual’s perceived role or their organisation’s culture resulting in an assessment of how relevant it is. Can reveal areas of “pushback” of an intervention that might otherwise not be apparent [51, 52].	• Views of the activities the participants were involved with. • Inclusion or exclusion of colleagues in the teams. • Roles they were asked to play.
Feasibility	Fit, practicality of the project.	• Fitting implementation team activities into current workload. • How useful or practical they perceived the activities to be.
Cost	Time burden and resource cost borne by participants. *Cost* usually refers to monetary cost or value for money but as HCP’s individual or group effort largely drove the interventions and they gave their time in kind, here we equated cost as value for contributed time [52].	• The amount of time and effort involved in the project.
Adoption	Extent to which participants changed their practice, or set an intention to do so, due to the project; here strongly associated with the primary outcome of referral rate.	• How has the participant’s practice changed (personal behaviour)? • How has their colleagues’ practice changed (corporate behaviour) due to the project interventions?

### Reflections

Health service researchers who facilitated the project (NT, JL) wrote structured reflections on their experience. Reflexivity of these pieces was influenced by their respective backgrounds: NT in health psychology; JL in nursing and biological science. NT had run previous TDFI projects in the UK but the methodology was new for JL. The reflection questions are provided in Additional file 2.

### Procedure

Invited participants were asked to give written consent for the interview to be recorded, and once de-identified, for their comments to be used in wider reporting. The interviewer was a researcher (TW) who had not been involved in the project. Interviews were recorded and transcribed. Reflection questions were emailed to and completed by JL and NT prior to exposure to interview data.

### Analysis

Interviews were audio recorded and transcribed verbatim. Inductive thematic analysis of both the transcribed interviews and reflections was undertaken to identify key themes across broad domain areas relating to participants’ experiences of the TDFI approach. A modified constant comparative method [20] was used to facilitate identification of themes. This involved two stages: [21] firstly, each interview was coded deductively by three researchers (JL and either TW or CZ) using the broad concepts of acceptability, appropriateness, feasibility, adoption, and cost. Secondly, two researchers (NT and JL) reanalysed the interviews to identify sub-categories under the broad concepts. The researchers met and compared codes to resolve disagreements, and add new codes, until consensus on themes was reached.

### Recommendations

The final themes and sub-themes were then further reflected on by experienced TDFI practitioners (NT, JL and DD) and using supporting data and literature [12, 22–38], provided a set of recommended refinements to the future use of TDFI in similar settings. These are summarised and described in more detail in the discussion.

## Results

**Fig. 2:**
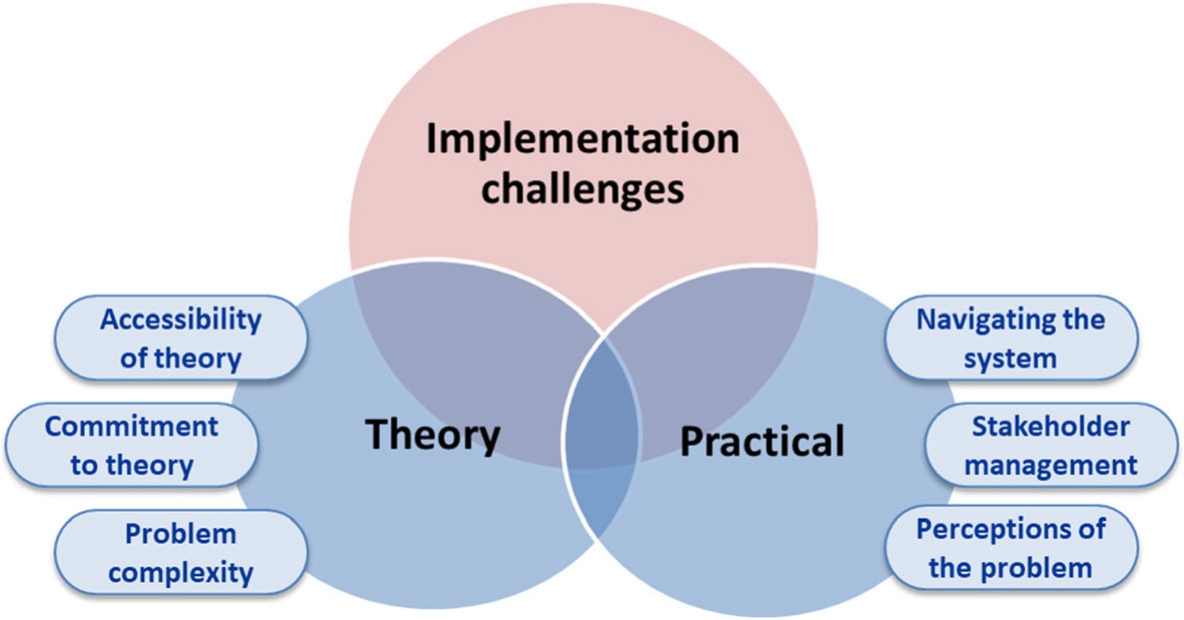
Implementation challenges affecting the perceptions of acceptability, appropriateness, feasibility, adoption, and cost of using the *Theoretical Domains Framework Implementation* approach

Nine participants (five from Hospital A and four from Hospital B) agreed to take part in the interviews. Participants included surgeons, pathologists, medical and radiation oncologists, genetic counsellors and administrative officers. All but two participants had more than 5 years’ experience in their roles. Reflections were written by the two health services researchers. Under the umbrella of ‘implementation challenges’ – affecting the perceptions of acceptability, appropriateness, feasibility, adoption, and cost of using the TDFI for improving referral rates of CRC patients with a high risk of LS – two main themes, each with a set of subthemes, were identified: 1) Theory related challenges, and 2) Practical challenges. Relationships of the themes and subthemes are shown in Table 2 and Fig. 2.

**Table 2:** Themes and subthemes relating to Theoretical Domains Framework Implementation (TDFI) challenges

Theme	Subtheme	Subtheme definition
Challenges related to using theory underpinning the TDFI approach	Accessibility of theory	Ease of which the theory can be understood and applied; access to support from theory experts.
	Commitment to theory	Issues relating to participants’ understanding of the value of theory in eliciting behaviour change, and subsequent adherence to the use of theory at the prescribed stages of the research project.
	Problem complexity	Issues around the processes leading to the outcome, here the processes leading to identification and referral of patients flagged at high risk of Lynch syndrome.
Practical (or context) challenges	Navigating the system (and system changes)	Issues around governance and ethical regulatory requirements; understanding of local politics and tacit behaviours/cultural factors Constant changes affecting teams and processes, such as introduction of new IT systems.
	Stakeholder management	Issues related to multiple stakeholders across different disciplines and departments.
	Perceptions of the problem	Issues around the perceived effort one should invest in the identified problem; lack of awareness about generalisability of solutions across contexts.

### Theory underpinning TDFI approach

Factors associated with the theoretical components of the TDFI approach – i.e., behavioural analysis for the identification of target behaviours for change [35], the theoretical domains [39], the mapping of behavioural change techniques [22, 34], and the operationalisation of these into practical strategies [2], presented challenges which affected its application.

### Complexity of the behaviour

One of the key components of the TDFI approach is a process mapping exercise with clinicians to understand and stimulate discussion about the key behaviours, resources, and interactions involved. The process map graphic is matched to audit data which highlights gaps in practice and aids in establishing a target behaviour for change. Within the broad concept of “appropriateness”, the process mapping exercise received both negative and positive comments. The map highlighted the complexity of the process, and made it difficult to narrow down the problem into a single behaviour enacted by individuals to target:

*I think that (the process map) it’s just too complicated. You need extremely simple triggers and to have so many different permutations and so many different avenues, you look at something like this and you just go, it’s too complicated. [Surgeon #2].*

*So, with some of the early process maps from Hospital A, there were a lot of revisions, or additions…it did obviously take a long time to eventually get to the final process maps…a lot of that had to do with the fact that once you start looking at this, and having enough people in the room and enough people arguing over, “No, no, no. This is what actually happens,” “No, no, no. That’s what actually happens,” …I don’t think I would necessarily have the time to apply it to every other problem, if you get my meaning. [Surgeon #1].*

Whilst this mapping exercise was seen by some as a challenging and time-consuming process, it was valued in terms of its usefulness for reaching consensus on the patient pathway and informing areas to target for change:

*The complex nature of the process was only realised fully after this [process mapping] exercise, making the areas to be selected for change difficult to agree on, and the process map itself seemed quite convoluted, which could have frustrated clinicians, particularly those who were not involved in creating it. [Researcher#2_structure reflection].*

*I think [the process map] is brilliant. Yeah… I definitely think it’s very useful. I think it’s clear. I mean everyone responds differently to images compared to text… It’s a nice flow, you can see exactly where a particular patient or specimen fits in. So yes, I’m all for process mapping. [Pathologist #1].*

### Accessibility of TDF

The TDF was designed to be accessible to people with no specialist knowledge of health psychology. The healthcare professionals exposed to the theory as part of the project design or intervention development were, however unable to comment on the theoretical components of this methodology, or did not know about/understand its multi-domain psychosocial and environmental approach to change (as opposed to raising awareness and changing attitudes):

*I don’t know. It’s hard for me to comment, I just don’t know enough on the literature of behavioural change…[Surgeon#2].*

*I probably have [heard of the Theoretical Domains Framework Approach] but I probably couldn’t articulate what it is to you. [Oncologist#2].*

*I think it needs to be a parallel systems approach rather not just a focus on behaviour…And I don’t think just having the attitudinal change or awareness is sufficient to produce the actual behavioural change. [Oncologist#2].*

### Commitment to theory

Another key facet of the TDFI is a commitment to using the underpinning theories. Theory informs the identification of key psychosocial and environmental barriers to change and, alongside clinicians, to direct the co-design of practical strategies to address these barriers using behaviour change techniques. However, once people learned of the problems or gaps in practice (e.g., from the process mapping exercise), there was a tendency to jump straight to possible solutions (prior to the theory-based barrier assessment and intervention design phases). This meant interventions often evolved along the way, based on intuition not theory. This abandonment of the theoretical framework also possibly diminished its value for participants:

*What was interesting was that it [the process mapping exercise] would almost give people a taste of, potentially, what could be fixed. And they were coming up with their solutions and implementing the change before they’d even worked through the system, which you can’t discredit it but I can see how it’s going to be frustrating for the psychological researchers who were trying to map this and how it all worked. It just adds a complex layer for you. [Genetics#1].*

*I reckon I could still sit down in a few hours and implement the strategies and just fix the problem just like that and not rely on anyone’s behavioural change, or whatever, just have just automatic triggers. [Surgeon#2].*

*Yeah, because you [the healthcare professionals] are not pure enough, you’re not – because you guys [the research team] are looking at it from a very pure point of view. [Oncologist#3].*

*[The behaviour change approach does make sense for a project like this] apart from us jumping the gun and coming up with implementations before we should’ve. [Genetics#1].*

### Practical challenges

Practical, or contextual factors associated with applying the TDFI approach – specifically working through the bureaucracy and general nuances of the system, system changes affecting implementation, stakeholder management, and perceptions of the issue, were highlighted by participants as problematic. Many of these issues were coded in the first round as lack of feasibility issues.

### Navigating the system

As a team of university-based researchers with behavioural and implementation science expertise, working as outsiders to drive an implementation project within a hospital was required for this project. Despite a range of relevant clinical connections made to develop the proposal and acquire funding for the work, participants in the project noticed and were frustrated by the challenges faced for the research team to navigate the system in relation to obtaining representation from all relevant stakeholders, managing the ethics/governance process, attending a multidisciplinary team meeting, or catching an opportune corridor conversation:

*…come through more roadblocks so it’s like you [the researchers] arrive like a tourist and you don’t speak the language and you just have some basic things and you say, well, you have to go to something 100 km away and you don’t know how the system works. [Oncologist#3].*

*Initially there was a lot of delays with getting approval to go ahead with the project, I believe. [Pathology#1].*

*So the investment for a while was - the time was just ticking on being wasted. You have this whole group of people employed ready to go and you had no – you couldn’t do anything, you’re sitting on your hands just waiting for the approval of things and that came quite late and it was a long time from the approval to the governance kick off…even [the research team] all twiddling their thumbs or when can we start, when can we start? Had lined everything up, all ready to go, I was just waiting to start and then it was just waiting and waiting and waiting. And then when it mattered then they had to, kind of, almost do it out of volunteer time because we, because it, rolled on; you’re collecting the data but, oh, our funding’s stopped. [Oncologist#3].*

*Great concept of implementation science project. However, there were some problems in terms of the recruitment of the colorectal team members being organised and notified late. They have no idea what is happening, who to approach, who are exactly in the team and who are the research team. [Oncologist#1].*

*It was interesting to see the dynamics of the Hospital B team and how they had to form two groups as there was no common time at which they could meet. It was endlessly frustrating trying to pin down a time to meet. [Researcher#1_structured reflection].*

These difficulties in navigating the system and the researchers attempting to drive the project from the outside meant that – despite advocacy for the strategies – intervention development and implementation was stalled, or in some cases did not happen at all within the allocated project timeframe:

*We had hoped for a letter with a clear section for genetics and a clear section for medical oncology, one for surgery and one for radiation to be generated on each patient. That we still haven’t moved very far forward with, and, unfortunately, I think, part of it just has to do with the politics of the [multidisciplinary team meeting] and the politics of letters at the moment. The second intervention was some type of failsafe in the rooms, and in our clinics, where, again, we could see that there’d been a flag from genetics, but a deliberate decision to bring it up at a later date, and, again, we haven’t, unfortunately, put that into practice yet either…it just reinforces to me that there’s still a few things that we need to do. [Surgeon#1].*

*I’ve got on the to do list to try and get this [electronic] referral in place, but unfortunately, it’s having to take a back seat to other… Well it’s key people are involved in other major, like not just one project, they’ve got major other projects with burning deadlines. [Genetics#1].*

*So very frustrating hearing what people planned to do but being unable to do it for them and watching the idea fade. Not having access to things like [the patient management software] to see how they work and what is visible to clinicians and what not would have been very helpful. [Researcher#1_structured reflection].*

### Stakeholder management

Factors relating to stakeholder management affected the overlapping concepts of acceptability, appropriateness and feasibility of the TDFI approach in this setting. These include the identification of all the relevant members of staff to be involved, the internal politics, hierarchies, perceptions of psychological safety, and potential conflict of interests arising for some individuals (e.g., who were investigators or implementation team members but who also found themselves involved in the practice being targeted for change):

*The main limitation comes predominantly because of the fact that there is such a large network of people that are involved in this process, there’s a lot of stakeholders, and it’s obviously quite difficult getting hold of the stakeholders in the same room, it’s difficult having people bounce ideas off each other, feeling safe bouncing those ideas in that kind of environment, there’s a lot of other agendas between clinicians, political, which are outside of, I guess, the project itself. [Surgeon#1].*

*I didn’t expect quite so much professional territory marking. The flat refusal to allow a senior nurse to refer to genetics seems purely political; slightly more understandable is the pathologists not wanting to make referrals themselves which sadly translated into “we won’t even make a recommendation”. [Researcher#1].*

*And so it’s kind of, you can get lost in the stronger personalities within that, so it’s having to keep being vocal about it within those meetings. [Genetics#1].*

*Your collaborators and your facilitators might be the target of your improvement itself which you don’t really know when you first start until a lot later then – and then you potentially you could feel used at that time because you have now gone from an insider to an outsider. So how do you handle that? And what your disclosures are when you collaborate with people in that sense that you might be the problem we might need to fix; how do you express that with a way that does not make people not want to deal with you? [Oncologist#3].*

### Perceptions of the problem

The broad themes of acceptability and appropriateness were also linked closely to the varied perceptions of the problem being addressed. Some believed it should be high on the public agenda because of the broader implications of this type of improvement for other areas of genetics, while others indicated there were bigger and more worthy problems to focus upon:

*I think it’s got a huge public health implication and highly relevant…but trying to improve their awareness of more than just Lynch Syndrome, I think they (surgeons, oncologists) often just think, oh Lynch Syndrome and FAP are the kind of key things that we’d (genetic specialists) be interested in, but, you know, trying to link in personal history of breast cancer with a pancreatic cancer, or you could think of a different, on the BRCA gene and things like that. [Genetics#1].*

*It was probably a lot of energy spent over a real problem but probably not the most important problem if - so to speak, so I don’t – to those people whose Lynch referrals increased it makes a big difference to them but overall the numbers were small and this is a large effort that required a lot of involvement of – there was a lot of energy that was put into it and it almost – you wish that it was being attached to something a little bit bigger. [Oncologist#3].*

*There are, certainly in my practice, there are a lot more urgent priorities that could be improved, whereas – and this is pretty small print stuff… will it make a lot of change? It might do for an individual too, but probably not a great amount of change, certainly for my practice and colorectal cancer in general. [Surgeon#1].*

## Discussion

We used qualitative data from semi-structured interviews and structured reflections to undertake a process evaluation of the application of the TDFI approach to address low referral rates for LS and to explore reasons for mixed results. Two main themes, each with three subthemes, were identified from the data to represent challenges associated with applying the TDFI approach. Here, we discuss the implications of these challenges and, alongside supporting data and literature, provide a suite of recommended refinements to the TDFI approach for use in implementation research.

The ‘complexity of the problem’ being addressed in this project was highlighted through the process mapping exercise which was undertaken as part of the behavioural analysis to identify a specific target behaviour for change (Step 2 of the TDFI approach). Despite working with experienced clinicians to understand the problem and develop the project submission, this only scratched the surface of the process. Therefore, when the implementation team meetings were held to break down the referral process with clinicians, the complexity of the process, and the actors and interdependencies involved, made it difficult to generate a simple process map that reflected enough detail to identify the gaps in practice. Furthermore, in order to generate a target behaviour for change – a key requirement of the TDF [39] – and understand the barriers to that behaviour, the team had to balance multiple considerations. To be inclusive of all involved staff, and be efficient with people’s time, the team elected to keep the statement of the target behaviour broad so that anyone involved in the referral process could answer the questionnaire from the perspective of their own individual role in ‘ensuring patients with a high risk of Lynch Syndrome are referred to genetic services’. Therefore, behaviours specific to different roles and the barriers to these behaviours were blurred and undefined or may have reflected a more general confusion over roles and responsibilities. As such, key interventions may have been missed. An alternative approach to this in the future could be to separate out specific target behaviours between staff groups performing different roles in the referral process.

With regards to ‘accessibility’, whilst the TDF was designed with an aim of being more accessible to people with no specialist knowledge of health psychology [39], the time and processes involved in understanding and applying the theory to design interventions should not be underestimated. Despite a range of presentations delivered by the research team in the participating organisations with information about the TDFI approach, and participation in implementation teams and/or focus groups to co-design interventions based on underpinning theory, participants involved in the project admitted a lack of understanding of behavioural change theory, or perceived that it was not necessary for eliciting change in the system. Without a clear understanding, clinicians may not have realised the potential value of the approach, and believed an intuitive approach to solutions was more worthwhile. Consequently, the traction and momentum needed for the application of the TDFI in the system may have been lost.

The lack of understanding, value, and traction of the TDFI are likely to have impacted on its intended ‘commitment to theory’. The involvement of individuals with key contextual knowledge are crucial for the co-design of practical and feasible strategies that are likely to be implemented, adopted, and sustained [31]. However, co-designing interventions using behavioural change theory requires, at least, a basic understanding of the domain and behaviour change technique meanings to develop practical strategies to address barriers. Covering this amount of material in a short focus group with time-poor clinicians is unfeasible. Furthermore, as demonstrated in our interviews, the health system often moves too fast for the strict application of theory: once clinicians learned of the problem that existed and could see the gaps in their practice mapped out in front of them from the process mapping exercise, the response was to jump straight in and “fix it.” [38] This meant that intervention strategies were being discussed prior to the formal design phase and even prior to barrier assessment, preventing compliance with the stipulated theoretical framework. Consequently, the potential impact of the intervention strategies may be diminished because they are not theory driven, and it is difficult to test the true impact of the framework, or attribute any effects on change to the theoretical components of the TDFI approach [37]. Whilst these implications may be frustrating for researchers attempting to discover the true benefits of a theoretical approach to change, these sentiments are likely not shared by those working within the system with, justifiably, more interest in simply improving practice. The benefits of this knowledge, nonetheless, would be realised by those individuals should evidence on well tested and effective strategies be available [28, 33, 40].

Difficulties of being external researchers included having no power to initiate interventions or to make changes, and having limited understanding of tacit behaviours and local politics. Moreover, being located off site gave no opportunity for incidental meetings (e.g., in the corridor or carpark), often cited as being beneficial to collaboration [30]. The pressure of service delivery meant that most participants had little time to attend project meetings and scheduling these was difficult. In several instances, two parallel, or multiple one-on-one meetings had to be arranged, changing direct multidisciplinary collaboration into brokered collaborations with the researchers as go-betweens.

From a practical perspective, ‘navigating the system’ was a key factor affecting the feasibility of the TDFI approach in this setting. Whilst the intention was to bring the theoretical expertise to the clinical context, the extent to which we as implementation researchers must understand the clinical field (e.g., genetics), how other clinical areas relate (e.g., colorectal surgery, pathology, oncology, genetic counselling), who the key people are, find convenient times to meet and discuss the project, and establish relationships to even get past the first hurdle of submitting a governance application, is time consuming and may even be perceived as inefficient. For example, the lengthy and bureaucratic governance process, a frequently debated topic in Australian research [41], took over 6 months. The negative impact of these delays on project momentum and clarity was very apparent to the research team at the time, and clearly perceived by the healthcare professional stakeholders. One alternative approach to mitigate some of these issues may be to consider the possibility of training and potentially seconding staff from within the system to deliver the TDFI approach as part of an implementation research project, with continuous guidance and support from the research team.

Another reason for mixed results related to ‘stakeholder management’ is that the research team had variable success in their roles as boundary spanners in this project. Boundary spanners have long been identified as key people who drive dissemination and build more cohesive teams to address change [26, 29, 32, 36, 42]. Researchers had two boundary spanning roles: bringing expertise of psychosocial barriers of change to the acknowledged common goal of increasing referrals for LS, and bringing together the different disciplines involved in the referral process. The second role was only partially successful. Building collaboration between different members of the multidisciplinary team was most successful at the start of the project as pathologists, geneticists, surgeons and oncologists met and pored over the various iterations of the process maps. As the project proceeded and scheduling group meetings became more difficult, rather than not meet at all, researchers compromised and met people face to face to keep up engagement and momentum. This did not assist in building a cohesive team and may have contributed to frustration with the process. Researchers were often put into go-between roles as direct multidisciplinary collaboration could not be achieved, and researchers lacked authority to make changes themselves.

Furthermore, this project revealed insights that alluded to the existence of an invisible line within the realms of implementation research; that is – healthcare professionals involved in the development of, or support for research projects to test improvement approaches in their health system may become the target of change, and the extent to which they realise this at the outset may impact on their reactions to and ongoing involvement in the implementation process [23]. A health system stakeholder initially recognising the opportunity to drive improvement through a formal research approach may, when their own practice receives scrutiny, experience feelings of vulnerability or embarrassment – factors that may not be conducive to continued support. These are extremely crucial and ethical considerations that should be raised, monitored, and managed by research teams prior to and throughout a piece of implementation research.

The ‘perceptions of the problem’ being addressed through this research differed between healthcare professional roles, and therefore the impact of opinions from those who did not perceive that improving the identification of LS patients was an appropriate priority may have impacted on the success of the project. We note that LS is a low incidence condition. The two hospitals saw around 200 CRC patients per year but only one or two patients per month were flagged as at high risk of LS. Nevertheless, the importance of tackling low referral rates is crucial. On an individual level these patients can be empowered to prevent, for themselves and their family members, a range of additional cancers to which they are predisposed [43]. A more global view is that our health system is being faced with an increasing number of low incidence hereditary conditions, which are being discovered every day [44]. The health system needs a way to manage these. If we can learn about how to improve clinical practice for one hereditary syndrome, there are likely to be transferrable strategies across others. For this we need evidence based, standardised, and systematic approaches to intervention design and implementation.

### Possible solutions for overcoming reluctance to use theory, and practical challenges

This analysis revealed several issues with the TDFI approach that may have contributed to the mixed results of the project. Potential modifications to the TDFI approach to address these issues include training individuals working within the health system, unpicking complex processes to define behaviours specific to different roles, and a greater recognition that health care settings are complex adaptive systems (Table 3).

**Table 3:** Subthemes and associated implementation outcomes and their suggested solutions

Subtheme	Implementation Outcomes	Suggested solutions
Accessibility of theory underpinning TDFI	Appropriateness	• Internal healthcare professional facilitators trained and supported by external TDFI experts
Commitment to use of theory	Acceptability Appropriateness	• Addressing more focussed behaviours • Internal healthcare professional facilitators trained and supported by external TDFI experts • Flexibility around quantitative and qualitative assessment of barriers • Rigorous research designs and process evaluations to assess application of theory and intervention fidelity
Problem complexity	Acceptability Appropriateness Cost	• Unpick complex processes to define behaviours specific to different roles
Navigating the system and system changes	Appropriateness Feasibility	• Internal facilitators trained and supported by outside TDFI experts • Understanding of the health system as a complex adaptive system • Process evaluations to unpick context based factors influences on intervention effects
Stakeholder management	Acceptability Appropriateness	• Internal facilitators trained and supported by outside TDFI experts
Perceptions of the problem	Acceptability Appropriateness	• Addressing more focussed behaviours • Internal facilitators trained and supported by external TDFI experts

*TDFI* theoretical domains framework implementation

People employed by the hospital or another health care organisation could be trained in the use of the TDFI approach, linked up with outside TDFI experts, and then facilitate the project themselves solving many of the barriers experienced here. An internal facilitator would better understand the context, local politics and tacit knowledge. They could connect with the relevant stakeholders and assess more readily what interventions are likely to work. The process of obtaining governance requirements for internal facilitators is simpler and more streamlined, again saving time and effort. Support and gentle insistence of the commitment to theory could be provided by the external experts [24]; uptake of this kind of approach could position participating health systems as leaders in implementation of evidence based policy and quality care [45].

To accurately assess barriers, the behaviours need to be tightly defined [46]. The recommendation here is to include an initial assessment of the process early in Step 2 of the TDFI to determine whether the target behaviour is discrete enough to provide clear barriers that can be addressed. Processes that include multiple behaviours performed by different stakeholders, especially if they occur across different departments (e.g., genetics, pathology and surgery) are likely to be too diffuse to yield useful barriers to inform intervention design. It may be that attempting to define behaviours for the purposes of a questionnaire to then determine key barriers, is too restrictive in some instances of evidence implementation, depending on the complexity of the process. Where complexity is an issue, it may be more useful to move straight to a theory guided focus group discussion about key barriers to performing behaviours. This would allow more flexibility in the discussion, and experts could then use this data to identify which barriers belong to the target behaviour for change. This approach may, however, lose some of the generalisability that a questionnaire can offer regarding barriers across an organisation, as well as the impact of reporting key barriers to clinicians from a quantitative perspective.

The TDFI approach does not make explicit the inherent complexity of the health care settings in which it is used. The final recommendation is to consider the features associated with complex adaptive systems [47] which can be drivers or barriers to the success of interventions [23, 48]. These features include: connections (or lack of connection) between often siloed departments, interdependencies and downstream effects of group decision-making by multidisciplinary teams, continuous learning from day to day practice and adaptation that incrementally occurs, the tendency for teams to self-organise, and groups’ need for sense-making before changing practices. Through rigorous research designs and in depth process evaluations, it will be more possible to unpick these system and cultural complexities, as well as the impact of theory and specific mechanisms of action associated with successful implementation approaches [24, 49].

### Strengths and limitations of the study

A strength of this process evaluation was that interviews were performed and co-analysed by a neutral third party. This was necessary as health services researchers running the evaluation project had built up relationships with the health professionals interviewed and could have influenced their responses. While qualitative data such as this provide a rich description of experience and should not be considered either representative nor generalisable, a larger sample of participants might have strengthened the research and raised additional issues. The main project was only conducted at two hospitals and had no matched controls. The complexity of the processes in oncology around screening and referral pathways and the unique cultural and political contexts at each make generalisability difficult. Recommendations point to ways of unpicking these contextual complexities to make the TDFI a more generalisable approach in similar settings.

## Conclusion

This study identified mixed perceptions of the acceptability, appropriateness and feasibility of the TDFI in this setting. Many factors were identified that potentially limited the impact of the TDFI approach, which are likely applicable to a range of theory-based implementation approaches. These included: the complexity of the referral process, the number of stakeholder groups involved, difficulties for external experts navigating and initiating change in the system, internal and external politics, a tendency for healthcare providers to implement intuitive solutions, and inconsistent application of theory. The study findings highlight challenges to maintaining ongoing commitment from champions initially enthusiastic about using the theory-based approaches to implement change, but also offer opportunities to improve acceptability, appropriateness and feasibility. Given the complexity of health care settings and differences in role-specific behaviours, we suggest that training an internal facilitator who possesses crucial knowledge of context and local politics may be more effective for the application of a theory-based implementation approach.

## Additional files

Additional file 1: Interview Schedule: Evaluation of the Lynch syndrome (LS) Project. (DOCX 16 kb)

Additional file 2: Structured reflection of the LS Project for health services researchers. (DOCX 15 kb)

## Abbreviations

CRC: Colorectal cancer
IPSBQ: Influences on Patient Safety Behaviours Questionnaire
LS: Lynch syndrome
TDF: Theoretical Domain Framework
TDFI: Theoretical Domain Framework Implementation

## References

[1] Taylor N, Lawton R, Moore S, Craig J, Slater B, Cracknell A (2014). Collaborating with front-line healthcare professionals: the clinical and cost effectiveness of a theory based approach to the implementation of a national guideline. BMC Health Serv Res.

[2] Taylor N, Lawton R, Slater B, Foy R (2013). The demonstration of a theory-based approach to the design of localized patient safety interventions. Implement Sci.

[3] Taylor N, Parveen S, Robins V, Slater B, Lawton R (2013). Development and initial validation of the influences on patient safety Behaviours questionnaire. Implement Sci.

[4] Colquhoun H, Leeman J, Michie S, Lokker C, Bragge P, Hempel S (2014). Towards a common terminology: a simplified framework of interventions to promote and integrate evidence into health practices, systems, and policies. Implement Sci.

[5] Moore G, Audrey S, Barker M, Bond L, Bonell C, Cooper C (2014). Process evaluation in complex public health intervention studies: the need for guidance. J Epidemiol Community Health.

[6] Craig P, Dieppe P, Macintyre S, Michie S, Nazareth I, Petticrew M. Developing and evaluating complex interventions: the new Medical Research Council guidance. BMJ. 2008;337. 10.1136/bmj.a1655 PMC2769032 18824488

[7] Long JC, Debono D, Williams R, Salisbury E, O’Neill S, Eykman E (2018). Using behaviour change and implementation science to address low referral rates in oncology. BMC Health Serv Res.

[8] Stoffel E, Mangu P, Gruber S, Hamilton K, MF LM (2015). Hereditary colorectal cancer syndromes: American Society of Clinical Oncology clinical practice guideline endorsement of the familial risk-colorectal cancer: European Society for Medical Oncology clinical practice guidelines. J Clin Oncol.

[9] Barrow P, Khan M, Lalloo F, Evans DG, Hill J (2013). Systematic review of the impact of registration and screening on colorectal cancer incidence and mortality in familial adenomatous polyposis and lynch syndrome. Br J Med Surg.

[10] Frayling I, Ward R (2014). Should we consider introducing systematic screening for lynch syndrome?. Cancer Forum.

[11] Ward RL, Hicks S, Hawkins NJ (2013). Population-based molecular screening for lynch syndrome: implications for personalized medicine. J Clin Oncol.

[12] Michie S, Johnston M, Abraham C, Lawton R, Parker D, Walker A (2005). Making psychological theory useful for implementing evidence based practice: a consensus approach. Qual Saf Health Care.

[13] Braithwaite J, Marks D, Taylor N (2014). Harnessing implementation science to improve care quality and patient safety: a systematic review of targeted literature. Int J Qual Health Care.

[14] Improvement Academy: Yorkshire asn Humber AHSN; [Available from: http://www.improvementacademy.org/]. Accessed 30 June 2018.

[15] Taylor N, Long JC, Debono D, Williams R, Salisbury E, O’Neill S (2016). Achieving behaviour change for detection and management of lynch syndrome using the theoretical domains framework implementation (TDFI) approach: a study protocol. BMC Health Serv Res.

[16] Long JC, Debono D, Williams R, Salisbury E, O'Neill S, Eykman E (2018). Using behaviour change and implementation science to address low referral rates in oncology. BMC Health Serv Res.

[17] Proctor E, Landsverk J, Aarons G, Chambers D, Glisson C, Mittman B (2009). Implementation research in menetal health services: an emerging science with conceptual, methodological, and training challenges. Adm Policy Ment Health.

[18] Proctor E, Silmere H, Raghavan R (2011). Outcomes for implementation research: conceptual distinctions, measurement challenges, and research questions. Adm Policy Ment Health.

[19] Proctor E, Silmere H, Raghavan R, Hovmand P, Aarons G, Bunger A (2011). Outcomes for implementation research: conceptual distinctions, measurement challenges, and research agenda. Admin Pol Ment Health.

[20] Glaser BG (2014). The constant comparative method of qualitative analysis. Soc Probl.

[21] Clay-Williams R, Baysari M, Taylor N, Zalitis D, Georgiou A, Robinson M (2017). Service provider perceptions of transitioning from audio to video capability in a telehealth system: a qualitative evaluation. BMC Health Serv Res.

[22] Abraham C, Michie S (2008). A taxonomy of behavior change techniques used in interventions. Health Psychol.

[23] Brainard J, Hunter PR (2016). Do complexity-informed health interventions work? A scoping review. Implement Sci.

[24] Davidoff F, Dixon-Woods M, Leviton L, Michie S (2015). Demystifying theory and its use in improvement. BMJ Qual Saf.

[25] Foy R, Ovretveit J, Shekelle PG, Pronovost PJ, Taylor SL, Dy S (2011). The role of theory in research to develop and evaluate the implementation of patient safety practices. BMJ Qual Saf..

[26] Greenhalgh T, Robert G, MacFarlane F, Bate P, Kyriakidou O (2004). Diffusion of innovations in service organizations: systematic review and recommendations. Milbank Q.

[27] Grol R, Bosch MC, Hulscher ME, Eccles MP, Wensing M (2007). Planning and studying improvement in patient care: the use of theoretical perspectives. Milbank Q..

[28] Grol R, Wensing M, Eccles M, Davis D. Improving patient care: the implementation of change in health care: Wiley; 2013.

[29] Kimberly J, Evanisko M (1981). Organizational innovation: the influence of individual, organizational, and contextual factors on hospital adoption of technological and administrative innovations. Acad Manag J.

[30] Knoben J, Oerlemans LAG (2006). Proximity and inter-organizational collaboration: a literature review. Int J Manag Rev.

[31] Leistikow IP, Kalkman CJ, de Bruijn H (2011). Why patient safety is such a tough nut to crack. BMJ.

[32] Long JC, Cunningham FC, Braithwaite J (2013). Bridges, brokers and boundary spanners in collaborative networks: a systematic review. BMC Health Serv Res.

[33] Marshall M, Pronovost P, Dixon-Woods M (2013). Promotion of improvement as a science. Lancet.

[34] Michie S, Richardson M, Johnston M, Abraham C, Francis J, Hardeman W (2013). The behavior change technique taxonomy (v1) of 93 hierarchically clustered techniques: building an international consensus for the reporting of behavior change interventions. Ann Behav Med.

[35] Michie S, Van Stralen MM, West R (2011). The behaviour change wheel: a new method for characterising and designing behaviour change interventions. Implement Sci.

[36] Rogers EM (1983). Diffusion of innovations.

[37] Scott I (2009). What are the most effective strategies for improving quality and safety of health care?. Intern Med J.

[38] Walsh J, McDonald KM, Shojania KG, Sundaram V, Nayak S, Davies S (2005). Closing the quality gap: a critical analysis of quality improvement strategies (Vol. 3: hypertension care).

[39] Michie S, Johnston M, Abraham C, Lawton R, Parker D, Walker A (2005). Making psychological theory useful for implementing evidence based practice: a consensus approach. BMJ Qual Saf.

[40] Foy R, Ovretveit J, Shekelle PG, Pronovost PJ, Taylor SL, Dy S (2011). The role of theory in research to develop and evaluate the implementation of patient safety practices. BMJ Qual Saf.

[41] Clay-Williams R, Taylor N, Braithwaite J (2018). Potential solutions to improve the governance of multicentre health services research. Med J Aust.

[42] Aldrich H, Herker D (1977). Boundry spanning roles and organizational structure. Acad Manag Rev.

[43] Lynch Syndrome Australia [Available from: https://lynchsyndrome.org.au]. Accessed 30 June 2018.

[44] Pogue RE, Cavalcanti DP, Shanker S, Andrade RV, Aguiar LR, de Carvalho JL (2018). Rare genetic diseases: update on diagnosis, treatment and online resources. Drug Discov Today.

[45] Ivers NM, Grimshaw JM (2016). Reducing research waste with implementation laboratories. Lancet.

[46] Atkins L, Francis J, Islam R, O’Connor D, Patey A, Ivers N, et al. A guide to using the theoretical domains framework of behaviour change to investigate implementation problems. Implement Sci. 2017;12(1). 10.1186/s13012-017-0605-9 PMC5480145 28637486

[47] Plsek PE, Greenhalgh T (2001). The challenge of complexity in health care. BMJ..

[48] Braithwaite J, Churruca K, Long JC, Ellis LA, Herkes J (2018). When complexity science meets implementation science: a theoretical and empirical analysis of systems change. BMC Med.

[49] Michie S, Fixsen D, Grimshaw JM, Eccles MP (2009). Specifying and reporting complex behaviour change interventions: the need for a scientific method. Implement Sci.

[50] Sekhon M, Cartwright M, Francis JJ (2017). Acceptability of healthcare interventions: an overview of reviews and development of a theoretical framework. BMC Health Serv Res.

[51] Klein K, Sorra J (1996). The challenge of innovation implementation. Acad Manag Rev.

[52] Proctor E, Brownson R, Brownson R, Colditz G, Proctor E (2012). Measurement issues in dissemination and implementation research. Dissemination and implementation research in health: translating science to practice.

