# Supplementary material for: Process evaluation of a behaviour change approach to improving clinical practice for detecting hereditary cancer
Source: BMC Health Serv Res. 2019 Mar 20;19:180. doi: 10.1186/s12913-019-3985-5 (PMC6425681; doi:10.1186/s12913-019-3985-5)
Supplement: Supplementary file 1 — Interview Schedule: Evaluation of the Lynch syndrome (LS) Project. (DOCX 16 kb) [file 12913_2019_3985_MOESM1_ESM.docx]

# Additional File 1

**Interview Schedule: Evaluation of the Lynch syndrome (LS) Project**

*Questions for Investigator and Implementation Team members*

Introduction: Thank you for agreeing to be interviewed. Everything you say will be treated as confidential. You will be referred to in any reporting as a generic role, e.g. “Implementation team member #2,” or “Investigator #5.” There is a survey we would like you to complete at the end. The interview and the survey together should take about 15-20 mins.

**Can I confirm that you are happy for this interview to be recorded and your de-identified comments used in any wider reporting (e.g. in journal articles)?**

You were part of the investigator/implementation team that ran the LS project. We are interested in knowing about your experience of that.

1. What were your general impressions of the project?
2. What was your role?
3. Can you tell me something about the team membership? Why do you think these people were chosen? *(Prompt: do you think any key opinion leaders, or people with particular experience or expertise, or any other stakeholders were left out?)*

*See table of team members and roles

1. The LS project used the Theoretical Domains Framework Implementation approach. Before your involvement in the LS, had you heard of the Theoretical Domains Framework approach? (*Yes/No answer is sufficient).*

*(Prompt: show Reference #1: 6 step TDFI flowchart)*

1. How well did the LS project match your expectations of how it would be - how did the LS project differ from any other quality projects you may have undertaken? We are thinking about things like the amount of time involved, amount you feel the team achieved, relevance to your practice or other work, having University researchers involved, or clinicians you hadn’t met
2. What input did you have in the development of the process maps used in the project? *(Prompt: for example, commenting on the early drafts*? *Show Reference #2a).* How useful do you think the process mapping was? What did the exercise tell you?
3. What did you think when you saw the audit results? *(Prompt: show Reference #2b or c. Audit numbers are in the circles).* What impact did seeing the process map with the matched audit data have on your perceptions of current practice?
4. The TDFI approach is based in behaviour change theory. Can you tell me what you know about behaviour change from your experience of the TDFI approach? Does the behaviour change approach make sense for a project like this? Did it result in feasible and appropriate strategies to increase referrals?

*(Prompt: did the domains of barriers make sense; did matching them to the appropriate Behaviour Change Techniques make sense?)*

*Show intervention table listing key barriers, techniques and implementation plan

1. Would you be interested in attending a training workshop and recurring tools on TDFI to learn more about evidence based implementation of guidelines, if one was made available? (Yes/No is sufficient)
2. As you probably know, the interventions were not all achieved. Some examples: there were disruptions around the move to the new Cancer services building, a new state-wide Genetic database was introduced, changes were made to the pathology database, there were delays with getting the forms on the oncology patient mnagement systems, and lack of capacity for some training sessions. Does this surprise you? How much do you think these things affected the rate of referrals?
3. As University researchers interacting with outsiders or external members to run an implementation project, it has been quite challenging. Whilst we have the research and scientific expertise, we don’t necessarily understand fully the entire context. What are your thoughts about involving hospital staff members (such as quality officers) being trained to use the TDFI approach so project facilitation can be done internally rather than by university researchers? Would more internal support have affected the success of the planned interventions?
4. How have you changed the way you do things as a result of the project?
5. What have you noticed about the way others do things differently as a result of the project? E.g. Pathology members or Hereditary Cancer Clinic staffs

*(Prompt: what is the change? Change in actions, documentation, attitudes or perceptions all of interest. Also includes changes to conducting research, QI issues elsewhere)*

1. Do you have any other comments you would like to make about your overall experience of working on the project?

Thank you very much for your time
