# Supplementary material for: Process evaluation of a behaviour change approach to improving clinical practice for detecting hereditary cancer
Source: BMC Health Serv Res. 2019 Mar 20;19:180. doi: 10.1186/s12913-019-3985-5 (PMC6425681; doi:10.1186/s12913-019-3985-5)
Supplement: Supplementary file 2 — Structured reflection of the LS Project for health services researchers. (DOCX 15 kb) [file 12913_2019_3985_MOESM2_ESM.docx]

# Additional File 2

**Structured reflection of the LS Project for health services researchers**

1. What were your general impressions of the project? (1 or 2 sentences)
2. What was your role?
3. Can you make some comments about the formation, membership and logistics of the implementation teams at each hospital? E.g. did we choose the right people, timing of meetings, etc.
4. The LS project used the Theoretical Domains Framework Implementation approach. Before your involvement in the LS project, had you heard of the Theoretical Domains Framework approach? (*Yes/No answer is sufficient).*
5. How well did the LS project match your expectations of how it would be? E.g. the amount of time involved, what the team achieved
6. Can you comment on the process mapping exercise? How useful do you think it was? How did clinicians respond to it?
7. What did you think when you saw the audit results? What impact did seeing the process map with the matched audit data have on clinicians’ perceptions of current practice?
8. The TDFI approach is based in behaviour change theory. Can you comment on what you have learnt about behaviour change from your experience of the TDFI approach? Does the behaviour change approach make sense for a project like this? Did it result in feasible and appropriate strategies to increase referrals?
9. The last audit showed that a clear increase in referrals was not achieved although parts of the process were changed. What is your take on this? What do you think is the reason that referrals have not increased despite our best efforts?
10. Can you comment on being an external facilitator able to advise but not actual do anything? Would more internal support have affected the success of the planned interventions?
11. How have you changed the way you run implementation projects as a result of the project?
12. Do you have any other comments you would like to make about your overall experience of working on the project?
